# Projection resolved optical coherence tomography angiography to distinguish flow signal in retinal angiomatous proliferation from flow artifact

**DOI:** 10.1371/journal.pone.0217109

**Published:** 2019-05-15

**Authors:** Alaa E. Fayed, Amani A. Fawzi

**Affiliations:** 1 Department of Ophthalmology, Feinberg School of Medicine, Northwestern University, Chicago, Illinois, United States of America; 2 Department of Ophthalmology, Kasr Al-Ainy School of Medicine, Cairo University, Cairo, Egypt; Boston Medical Center, Boston University School of Medicine, UNITED STATES

## Abstract

**Purpose:**

To investigate whether hyperreflective foci (HRF) exhibit flow projection artifact on OCTA, and study the efficacy of commercial projection artifact removal software (PAR-OCTA, Optovue, Inc), and a custom projection resolved OCTA (PR-OCTA) in distinguishing artifacts from true flow in retinal angiomatous proliferation (RAP).

**Methods:**

The study included five eyes with HRF representing pigment migration in dry age-related macular degeneration (AMD), five eyes with leaking treatment-*naïve* RAP, and ten eyes with diabetic hard exudates. We examined flow signal on OCTA cross-sections using PAR, and performed PR-OCTA to study the effect of increasingly stringent projection removal thresholds. Flow signal intensity was analyzed and quantified using imageJ (NIH, Bethesda, MD, USA), by calculating the percentage of red pixels (R) representing flow, compared to green (G) and blue (B) pixels.

**Results:**

PAR-OCTA cross sections revealed persistent flow signal in all HRF, including RAP, hard exudates and pigment migration. In RAP, PR-OCTA detected intransigent flow, irrespective of the flow removal threshold. Mean R in the five RAP lesions remained higher than mean G and B at the most stringent PR-OCTA threshold (40.96% vs 29.52 and 29.52%, respectively), denoting persistence of flow. In contrast, increasing the PR-OCTA threshold in pigment migration and hard exudates removed the flow signal, with a statistically significant decrease in mean R with increasing threshold. (p = 0.017 and 0.0029, respectively)

**Conclusion:**

Commercial PAR-OCTA is not completely effective at removing artifactual flow in hard exudates and HRF related to pigment migration. Custom built PR-OCTA, using a sliding scale of threshold, allowed us to distinguish true flow in RAP from artifactual flow in avascular HRF. Further studies are needed to validate the optimum threshold for projection artifact removal, which would preserve true flow in RAP and the small intraretinal capillaries.

## Introduction

Retinal angiomatous proliferation (RAP) is a unique form of neovascular age-related macular degeneration (AMD), first suggested by Hartnett et al. [1], as an abnormal complex of deep retinal vessels accompanied by retinal pigment epithelial (RPE) detachments. The term RAP was introduced by Yannuzzi et al. [2] to suggest the retinal origin of the neovascular fronds, a finding that was later confirmed by various groups using high resolution imaging, as well as histopathological studies. [3–5]

Until recently, the preclinical stages of RAP had not been accessible to imaging studies. Bhavsar et al. [6] were first to demonstrate abnormal flow on optical coherence tomography angiography (OCTA) that later developed into clinically active, leaking RAP lesions. More recently, a preclinical "nascent" stage of RAP was reported by Sacconi et al. [7], characterized by subtle intraretinal hyperreflective foci (HRF) without intraretinal fluid on structural OCT. These HRF demonstrated hyperfluorescence on fluorescein and indocyanine green angiography, as well as detectable flow on OCTA. Interestingly, the authors found that 20% of these HRF did not progress into active RAP in subsequent visits during one year follow up. They concluded that the diagnosis of nascent RAP should warrant close follow up with OCTA to document the downward growth of the lesion towards the sub-RPE space, along with intraretinal fluid on OCT signaling the progression into the actively leaking stage. [7]

Despite the important insights provided by Sacconi et al. [7], there remains the issue of projection artifacts, which are most prominent in hyperreflective structures. [8, 9] In order to remove projection artifacts, Zhang et al. [10] developed an image-processing algorithm called projection resolved OCTA (PR-OCTA). This algorithm has been used to demonstrate the three macular capillary plexuses of the inner retina [11], show early flow prior to clinical diagnosis of RAP lesions [6], identify the microvascular changes associated with paracentral acute middle maculopathy and acute macular neuroretinopathy [12] and to illustrate the clinical implication of the three dimensional complexity of choroidal neovascularization. [13]

Notably and relevant to RAP, HRF are not an exclusive feature of nascent RAP. They have been described in various other retinal and choroidal disorders, including diabetic macular edema [14, 15] and retinal vein occlusion [16], where they are hypothesized to represent extravasation of lipid and other blood components. HRF are also documented in various AMD forms including acquired vitelliform lesions [17], intermediate AMD and dry forms of late AMD [18], where they are thought to represent RPE proliferation and intraretinal migration. [19, 20] Based on these findings, HRF in the context of AMD may represent a spectrum of pathologies, including treatment-requiring RAP lesions, as well as the more innocuous migrating RPE cells.

In the current study, we investigated whether eyes with HRF on OCT would exhibit flow projection artifact on OCTA. We compared flow signal in eyes with actively leaking RAP to two groups of eyes, representing HRF without underlying vascular pathology; a group with RPE migration in the setting of intermediate to late dry AMD, and another with hard exudates in the setting of diabetic retinopathy. We then investigated the role of PR-OCTA in removing flow projection artifact generated by these non-vascular HRF lesions.

## Patients and methods

This was a retrospective analysis of OCTA images performed on patients with either AMD or diabetic retinopathy recruited in the Department of Ophthalmology at Northwestern University in Chicago, Illinois between June 2017 and January 2019. The study was approved by the institutional review board of Northwestern University, followed the tenets of the Declaration of Helsinki, and was performed in accordance with Health Insurance Portability and Accountability Act regulations. Written informed consent was obtained from all participants.

### Study sample

**Fig 1:**
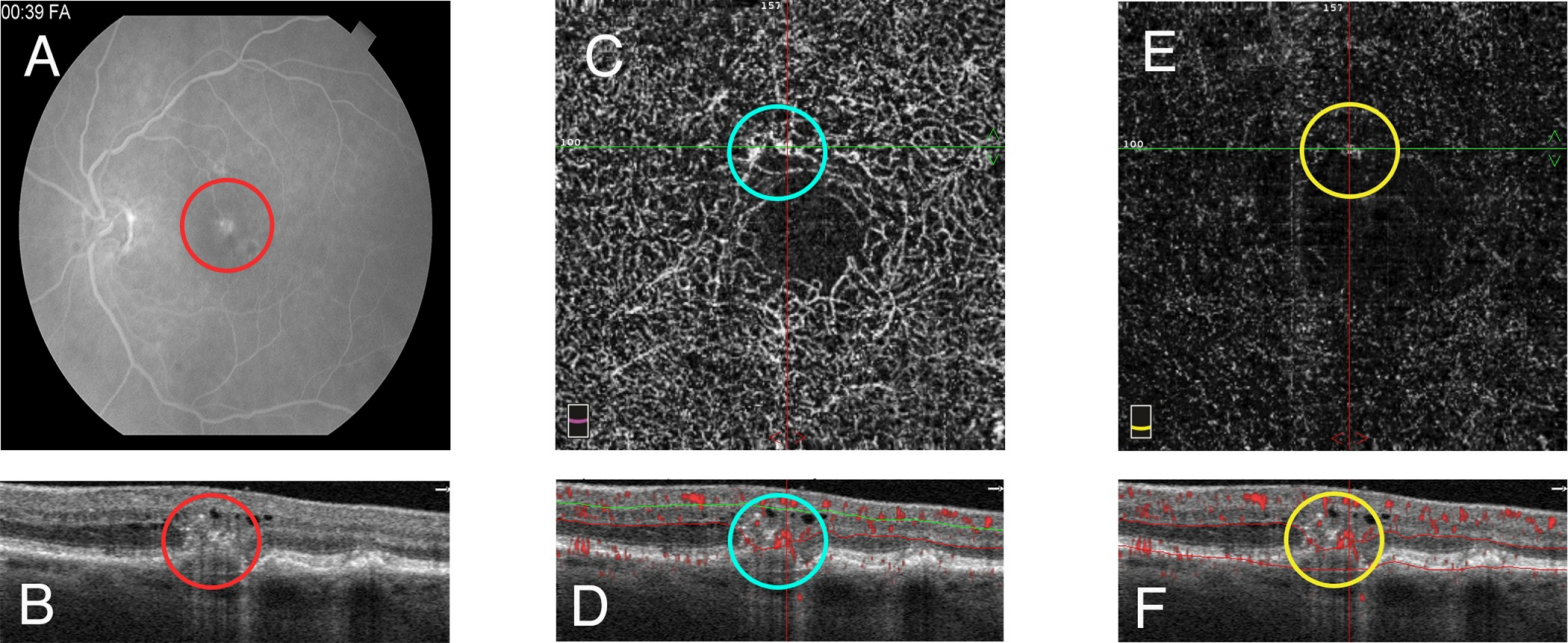
Multimodal imaging of retinal angiomatous proliferation (RAP). A. Fluorescein angiogram during the late venous phase shows a hyperfluorescent leaking "hot spot" superior to the foveal avascular zone, corresponding to the RAP lesion. (red circle) B. Optical coherence tomography (OCT) cross section showing a hyperreflective intraretinal lesion. (red circle) C. *En face* optical coherence tomography angiography (OCTA) of the deep capillary plexus (DCP), shows an abnormal vascular structure superior to the foveal avascular zone. (cyan circle) D. OCTA cross section representing the DCP slab in (C), segmented with an upper boundary 30 μm below the inner plexiform layer (IPL), and a lower boundary set at 10 μm below the outer plexiform layer (OPL), shows flow signal starting within the hyperreflective lesion arising from the DCP and traversing the outer nuclear layer (ONL) to reach the RPE. (cyan circle) E. *En face* OCTA of the outer avascular retina, reveals a downward-growing extension of RAP from the overlying DCP. (yellow circle) F. OCTA cross section representing the outer slab in (E), segmented with an upper boundary set 10μm below the OPL and a lower boundary set at 10 μm above Bruch's membrane, shows flow signal corresponding to the RAP lesion. (yellow circle).

The diagnosis of RAP was based on previously described spectral domain OCT findings [21], including intraretinal HRF and fluid, with leakage confirmed by fluorescein angiography. All eyes were treatment-naïve at the time of diagnosis (Fig 1). In eyes with diabetic retinopathy and hard exudates, we only included treatment-naïve eyes with hard exudates in the avascular outer nuclear layer (ONL) in order to avoid other possible confounding structures such as microaneurysms, which may manifest as HRF in the vascularized layers. For patients with AMD, we included treatment- naïve eyes with intermediate or late dry AMD, exhibiting areas of RPE migration appearing as intraretinal HRF without intraretinal fluid or evidence of progression over the following period of 6 months. The diagnosis for these patients was made based on clinical assessment by a retina specialist (A.A.F).

Only eyes that had OCTA images without significant movement or shadow artifacts, a quality index (Q) of 6 or more and a signal strength index (SSI) above 50 were considered eligible. Exclusion criteria were eyes with other retinal or choroidal diseases that may confound our results and eyes that have received intravitreal pharmacotherapy of any form, retinal laser or pars plana vitrectomy. We excluded eyes with astigmatism more than 3 diopters, high refractive errors (more than 6 diopters), or cataract graded above nuclear opalescence grade three or nuclear color grade three, to avoid optical artifacts that potentially may compromise OCTA image quality. Electronic medical records were reviewed to extract demographic and clinical information.

### OCT angiographic imaging and image processing

Patients underwent imaging using RTVue-XR Avanti device (Optovue Inc., Fremont, California, USA), with split-spectrum amplitude-decorrelation angiography (SSADA) software (version 2017.1.0.151). [22] This instrument has an A-scan rate of 70,000 scans per second and uses a light source centered at 840 nm and a bandwidth of 45nm. Two consecutive B-scans (M-B frames), each containing 304 A-scans, were captured at each sampling location and SSADA was used to extract OCTA information. 3D Projection artifact removal (PAR) technology by Optovue was used to obtain 3x3 mm scans centered on the fovea. [23] This algorithm is also based on the study by Zhang et al. [10] PAR-OCTA operates based on the premise that projection artifacts generally occur at a location with real OCTA signals anterior to it and when the location itself has strong OCT reflectance signal. [24] The algorithm utilizes information from the OCT and OCTA volume to differentiate in situ OCTA signal from projection artifacts based on OCTA and OCT intensity profiles anterior to and at the voxel of interest. [24, 25] In addition to the in situ OCT intensity and OCTA intensity, the Optovue method utilizes further information including depth cumulative OCTA intensity along the axial direction to differentiate in situ OCTA signal from projection artifacts. The projection artifacts, instead of being suppressed to zero intensity, are suppressed to have signal intensities at the background noise level. [25] Cross-sectional OCT scans with angiographic flow overlay were used to identify flow signal in the various types of HRF.

### PR-OCTA post-processing

We implemented a version of the PR-OCTA algorithm previously described by Zhang et al. [10] in a custom MATLAB (Mathworks 2015, Natick, MA, USA) program. The authors reported that the OCTA projection tail artifacts have lower decorrelation values than the overlying real vessels. The algorithm removes projection artifacts by searching for and preserving consecutively increasing decorrelation peaks (real flow) along each A-line. The OCTA values at the peak positions are kept, whereas the remaining lower peak (artifact flow) pixels in the A-scan are set to zero, resulting in the removal of projection artifacts. The threshold used to remove these lower OCTA values can be manipulated to study the intensity of the different flow artifacts. The decorrelation value generated by moving red blood cells in the superficial retinal capillaries in an eye is used to set the value of the maximum threshold of “1.0” in that eye. Using a threshold higher than 1.0 would therefore remove flow signal representing real blood flow. By setting the PROCTA threshold to progressively increasing fractions of this benchmark, we were able to methodically observe the changes in flow signal with increasing the PROCTA threshold. PR-OCTA scans were obtained at increasing thresholds and compared to corresponding built-in PAR-OCTA (Optovue, Inc) scans to compare the efficiency of these algorithms at eliminating flow projection artifacts. The same identical B scan obtained from the same macular cube was used for each eye to assess the presence or absence of flow signal, as well as to quantify pixels in the region of interest (ROI), to allow a fair and adequate comparison.

### Flow signal interpretation

In order to ensure an objective and unbiased approach to the interpretation of the flow signal presence, as well as any change in its intensity as a result of the software algorithm, we devised a pixel-counting algorithm. All images obtained by PAR-OCTA and the thresholded PROCTA algorithm were converted to an 8-bit RGB version, using the "image" tab in imageJ (NIH, Bethesda, MD, USA). The basis for this method is that each pixel in an 8-bit RGB image is represented as a combination of the three primary colors: Red, Green and Blue, each with a value from 0 to 255. [24, 25] Therefore, a pixel with RGB values (0,0,0) has a black color, whereas a pixel with values (255,255,255) is white. More pertinent to flow signal, if a pixel has more R than G or B, then it would be a shade of red, representing flow. In order to compare flow signal for the PAR software and the PR-OCTA algorithm, the ROI with flow signal was selected in cross-sectional OCTA scans, and flow signal analysis was performed using the "color histogram" feature in imageJ. Flow signal intensity was quantified by calculating the percentage of red pixels (R) representing flow in the ROI, compared to green (G) and blue (B) pixels. Higher R, compared to G and B, in any of the ROIs indicates an abundance of red, consistent with the presence of flow. Equal percentage of all 3 components suggests the disappearance of red in the ROI, ie: absent flow signal.

### Statistics

We performed statistical tests with SPSS version 21 (IBM SPSS Statistics; IBM Corporation, Chicago, IL). The percentage of red pixels (R) in eyes with migrating RPE and eyes with hard exudates was compared between PR-OCTA thresholds 0.3 and 0.7 using paired samples t tests. A P-value of less than 0.05 was considered statistically significant.

## Results

Five eyes with actively leaking RAP, five eyes with RPE migration in the setting of dry AMD and ten eyes with diabetic retinal hard exudates in the ONL were included (Table 1). All eyes showed OCT evidence of intraretinal HRF. Analysis of multiple OCTA cross-sections with angiographic overlay using the commercial PAR software revealed flow signal within the HRF in all eyes, regardless of the diagnosis.

**Table 1:** Mean percentages of red, green and blue pixels in the PAR-OCTA and PR-OCTA scans of the three study groups.

*Study group*					*Paired samples* *t test* (p value)
***Retinal angiomatous proliferation group***					
	*PAR-OCTA*	*PR-OCTA 0*.*5*	*PR-OCTA 0*.*7*	*PR-OCTA 1*.*0*	
Red pixels Mean % (SD)	40.958 (2.822)	40.769 (3.344)	38.417 (2.571)	37.184 (2.484)	-
Green pixels Mean % (SD)	29.521 (1.411)	29.689 (1.754)	30.852 (1.218)	31.399 (1.213)	-
Blue pixels Mean % (SD)	29.521 (1.411)	29.542 (1.598)	30.731 (1.368)	31.417 (1.28)	-
***Hard exudate group***					
	*PAR-OCTA*	*PR-OCTA 0*.*3*	*PR-OCTA 0*.*5*	*PR-OCTA 0*.*7*	
Red pixels Mean % (SD)	36.424 (2.417)	**37.474 (2.197)**	35.466 (2.048)	**33.47 (0.199)**	**0.0029**
Green pixels Mean % (SD)	31.788 (1.208)	31.345 (1.044)	32.25 (1.113)	33.344 (0.061)	-
Blue pixels Mean % (SD)	31.788 (1.208)	31.181 (1.199)	32.284 (1.025)	33.186 (0.169)	-
***Retinal pigment epithelial migration group***					
	*PAR-OCTA*	*PR-OCTA 0*.*3*	*PR-OCTA 0*.*5*	*PR-OCTA 0*.*7*	
Red pixels Mean % (SD)	37.89 (1.982)	**36.031 (1.093)**	35.03 (1.579)	**33.425 (0.121)**	**0.017**
Green pixels Mean % (SD)	31.055 (0.991)	31.801 (0.518)	32.48 (0.861)	33.283 (0.074)	-
Blue pixels Mean % (SD)	31.055 (0.991)	32.168 (0.637)	32.49 (0.765)	33.292 (0.05)	-

RAP = Retinal angiomatous proliferation, PAR-OCTA = Projection artifact removal-optical coherence tomography angiography, PR-OCTA = Projection resolved optical coherence tomography angiography, SD = Standard deviation, HE = Hard exudates, RPE = Retinal pigment epithelium, 0.3, 0.5, 0.7 & 1.0 = PR-OCTA thresholds.

**Fig 2:**
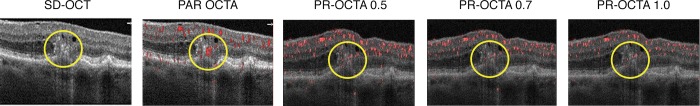
Projection-resolved optical coherence tomography angiography (PR-OCTA) of retinal angiomatous proliferation (RAP). Spectral domain-OCT (SD-OCT), projection artifact removal (PAR) OCTA (Optovue, Inc.) and PR-OCTA scans at thresholds of 0.5, 0.7 and 1.0 of the same eye in Fig 1. The RAP lesion (yellow circle) shows persistent flow signal despite increasing PR-OCTA thresholds. Red pixel percentage decreased between PR-OCTA thresholds 0.5, 0.7 and 1.0 from 42.5% to 39.9% then 37.7%, but remained higher at 1.0 than green and blue pixels (30.9% and 31.3%, respectively).

**Fig 3:**
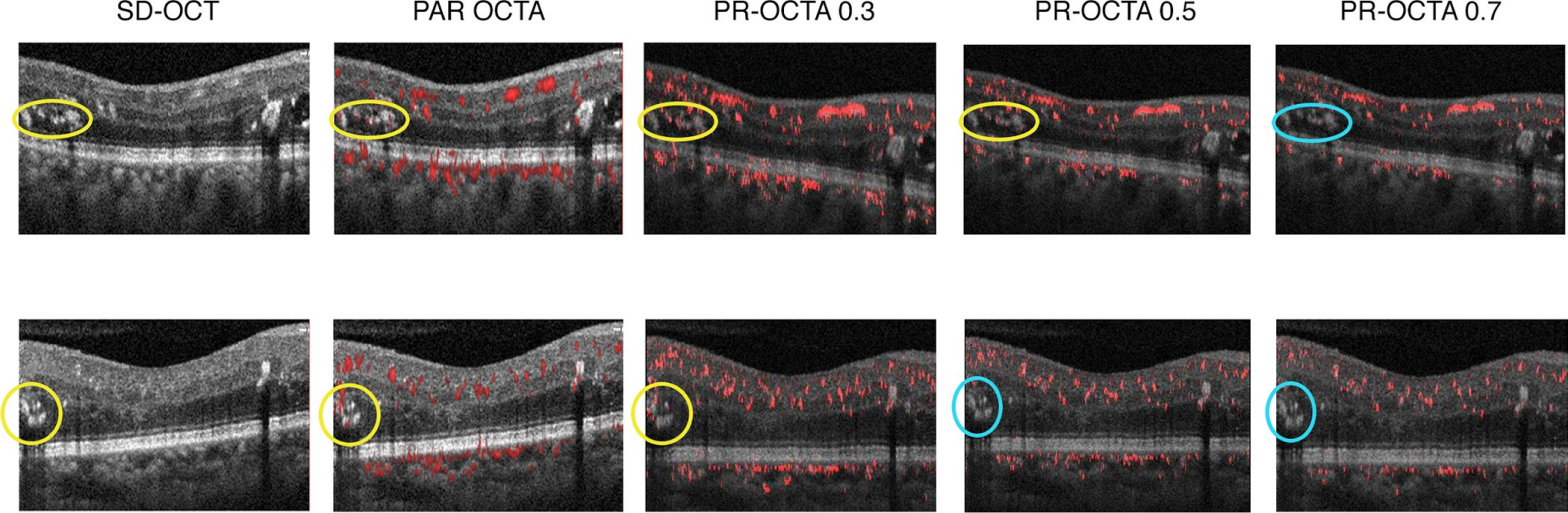
Projection-resolved optical coherence tomography angiography (PR-OCTA) of hard exudates. **Top row:** Spectral domain-OCT (SD-OCT), projection artifact removal (PAR) OCTA and PR-OCTA scans at thresholds of 0.3, 0.5 and 0.7 of case 13. The flow artifact signal is seen in PAR-OCTA in the hard exudate (yellow ellipse). There is gradual decrease in the flow signal intensity with increasing PR-OCTA threshold from 0.3 to 0.5 (yellow ellipses), manifested by a decrease in red pixel percentage from 38.4% to 34.5%. The flow signal disappears at a threshold of 0.7 (cyan ellipse), where the red pixel percentage is equal to green and blue pixels (33% each), thus confirming it is projection artifact and not true flow. **Bottom row:** SD-OCT, PAR-OCTA and PR-OCTA scans at thresholds of 0.3, 0.5 and 0.7 of case 17. The flow artifact is seen in PAR-OCTA in the hard exudate (yellow circle). The flow artifact in this eye disappears at PR-OCTA threshold 0.5 (cyan circle) compared to 0.7 in the top row. The red pixel percentage drops from 35.9% in 0.3 to 33% in 0.5, to be equal to that of green and blue, confirming its disappearance.

In the RAP group, PR-OCTA showed persistent flow signal in the RAP lesions, regardless of the PR-OCTA threshold used to eliminate projection artifacts (Fig 2). Mean R in the five RAP lesions at the most stringent PR-OCTA threshold remained higher than mean G and B (40.96% versus 29.52 and 29.52%, respectively), denoting persistence of flow. In the hard exudates group, there was a gradual decrease in the intensity of the flow projection with increasing PR-OCTA thresholds. Threshold values from 0.1 to 0.4 failed to remove flow projection artifact. Of the 10 eyes, only 1 (10%) showed complete resolution of the flow projection artifact at a threshold of 0.5. The remaining 9 eyes (90%) required a threshold of 0.7 to completely eliminate projection artifact. (p = 0.0029) (Fig 3)

**Fig 4:**
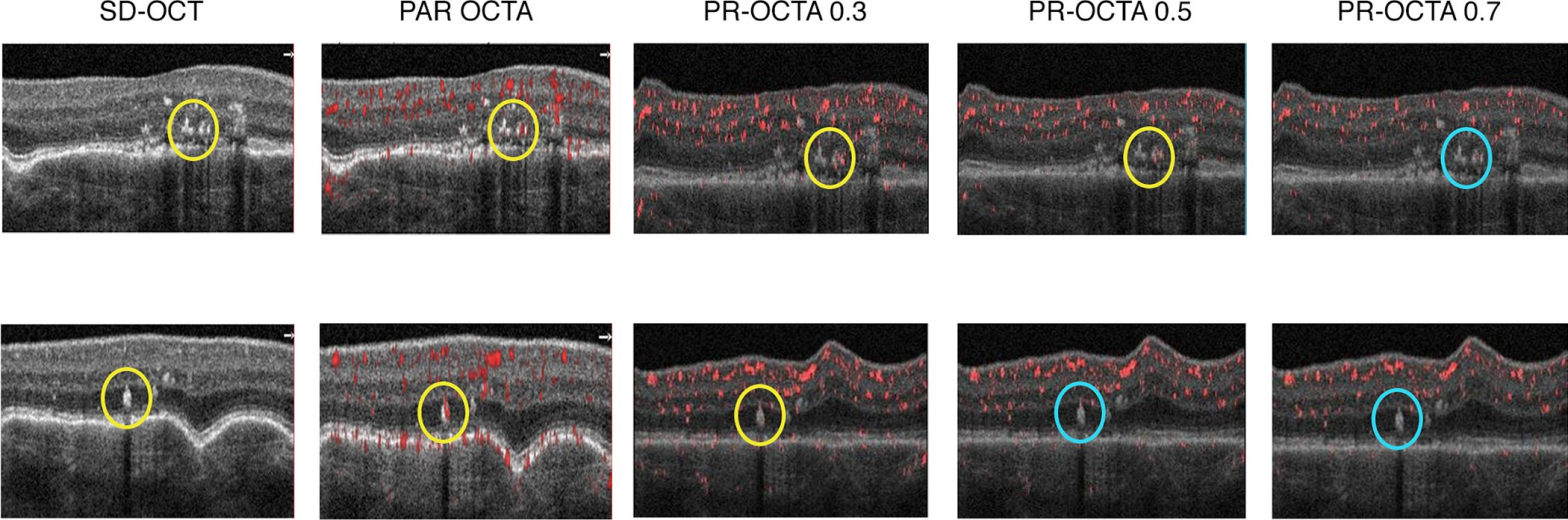
Projection resolved optical coherence tomography angiography (PR-OCTA) of migrating intraretinal pigment epithelium (RPE). **Top row:** Spectral domain OCT (SD-OCT), projection artifact removal (PAR) OCTA and PR-OCTA scans at thresholds of 0.3, 0.5 and 0.7 of case 6. The flow signal is seen in PAR-OCTA in the migratory RPE cells (yellow circle). There is gradual decrease in the flow signal intensity with increasing PR-OCTA threshold from 0.3 to 0.5 (yellow circles), manifested by a decrease in red pixel percentage from 36.2% to 34.3%. The flow signal disappears at 0.7 (cyan circle), where the red pixel percentage is equal to green and blue pixels (33% each), confirming it is projection artifact and not true flow. **Bottom row:** SD-OCT, PAR-OCTA and PR-OCTA scans at thresholds of 0.3, 0.5 and 0.7 of case 9. The flow artifact signal is seen in the HRF in PAR-OCTA (yellow circle). The flow artifact in this eye disappears at PR-OCTA threshold 0.5 (cyan circle) compared to 0.7 in the top row. The red pixel percentage drops from 34.7% in 0.3 to 33% in 0.5, to be equal to that of green and blue, confirming its disappearance.

The RPE migration group also showed a gradual diminution of flow signal with increasing threshold values. As with hard exudates, thresholds of up to 0.4 were insufficient to eliminate the flow signal. Two of the 5 eyes (40%) had complete resolution of the flow projection artifact at a threshold of 0.5. The remaining 3 eyes (60%) required a threshold of 0.7. (p = 0.017) (Fig 4). The mean percentages of R, G and B are highlighted in Table 1.

## Discussion

In this study, we demonstrate that HRF in the setting of intraretinal pigment migration and hard exudates are associated with flow artifact on OCTA that persist despite implementing commercial projection artifact removal software. We also show the ability of custom-built software to distinguish these projection artifacts from true flow in actively leaking RAP lesions. The pixel counting method allowed us to objectively quantify flow signal providing an unbiased approach to flow signal interpretation.

The ability of OCTA to produce high-resolution angiographic images of the retinal vascular plexuses has allowed a better demonstration of RAP as a vascular complex arising from the deep retinal capillary plexus. [26] Bhavsar et al. [6] demonstrated the presence of flow in an abnormally dilated single vessel in the deep capillary plexus on OCTA, five months prior to the development of intraretinal hemorrhage and active leakage on fluorescein angiography at the site of a RAP lesion. More recently, Sacconi et al. [7] identified the preclinical stages of RAP lesions and showed that HRF were present at baseline in all cases that later showed signs of activity. Using OCTA, the authors demonstrated that HRF sprout as vascular tufts at the deep capillary plexus, with detectable flow. Notably, the presence of HRF on SD-OCT is well documented in the setting of pigment migration in AMD [18–20], specifically also in eyes with RAP. [27] In fact, Sacconi et al. [7] also reported the presence of HRF from RPE migration in their group of patients with nascent RAP, and noted that HRF related to pigment migration were more common than those representing true nascent RAP lesions. Given the co-existence of HRF in eyes with RAP, and in view of our findings of artifactual flow in migrating RPE cells despite commercial projection removal software in OCTA, there is a pressing need to improve OCTA projection removal software.

Although OCTA is widely used to demonstrate various retinal and choroidal vascular pathologies, projection artifacts continue to plague the accurate interpretation of this imaging modality, prompting the development of various projection removal software. [8, 9, 28, 29] One option, PR-OCTA, was developed by Zhang et al. [10] to remove prominent decorrelation tails caused by flow projection artifacts. Using PR-OCTA, our study showed that flow signal inactively leaking RAP lesions was resilient, while HRF representing pigment migration showed gradually diminishing flow signal with higher PR-OCTA thresholds until flow artifact was eliminated at a threshold of 0.7. The avascular nature of the RPE cells produced a decorrelation value that is consistently lower than that caused by real blood vessels. Interestingly, our results show that the threshold required to remove projection artifact generated by pigment and exudate is not consistent, suggesting that these HRF are associated with variable reflectance. A threshold of 0.5 was sufficient to eliminate artifactual flow projection in 40% of the eyes with migrating pigment cells, but only in 10% of eyes with hard exudate. The source of these discrepancies is not clear to us at this point, however our results suggest that a threshold of 0.7 may be adequate to ensure removal of projection artifacts related to intraretinal pigment and hard exudate.

Our study was limited by its retrospective nature, as well as the relatively small number of eyes, which was imposed by our use of strict criteria to ensure the quality of the included images. Future studies with a larger study sample may shed light on the ability of PR-OCTA to mitigate flow projection artifact on a wider scale of patients. Our study is also limited by the lack of eyes with nascent RAP to validate the ability of PR-OCTA to interpret the nature of HRF in that setting. The strengths of our study include the introduction of HRF of various types such as migrating RPE and hard exudates in the ONL, which demonstrates the global impact of flow projection artifacts in these HRF, as well as the ability of PR-OCTA to mitigate them. In order to address any possibility of excessive removal of signal representing true flow by the PR-OCTA algorithm, we included eyes with RAP lesions. In these eyes, we confirmed the vascular nature of the lesions by fluorescein angiograms, allowing them to serve as a “positive control” group for the effect of the algorithm. Our results show that the algorithm could not remove RAP lesions, representing true blood flow, regardless of the threshold used. This confirms that the algorithm does not remove flow signal representing true flow. Another strength is the introduction of an objective pixel counting method to demonstrate flow signal presence and intensity, providing a quantitative and objective interpretation. The approach accounts for both the structural data provided by OCT, and the flow metrics demonstrated by OCTA to interpret flow signal at HRF, rather than rely on software-generated pseudo-colored outputs.

In conclusion, despite commercial projection removal software, OCTA is susceptible to artifactual flow signal in HRF caused by RPE migration in eyes with dry AMD, as well as hard exudates. We used an objective pixel-counting tool and PR-OCTA to demonstrate the resilience of real flow signal in RAP lesions, and the ability to eliminate artifactual flow caused by RPE migration and hard exudates. Future studies with a larger number of eyes is needed to investigate our proposed PR-OCTA threshold of 0.7 as potentially useful for removing pigment and exudate related projection artifact, while preserving real flow signal in RAP lesions.

## Supporting information

S1 Dataset Excel sheet describing the red, green and blue pixel values and means in the 3 groups of eyes: eyes with retinal angiomatous proliferation (RAP), eyes with hard exudation and eyes with migrating retinal pigment epithelial (RPE) cells. (XLSX)

## References

[1] Hartnett ME, Weiter JJ, Garsd A, Jalkh AE. Classification of retinal pigment epithelial detachments associated with drusen. Graefes Arch Clin Exp Ophthalmol. 1992;230(1):11–9. 1547961 10.1007/BF00166756

[2] Yannuzzi LA, Negrão S, Tomohiro I, Carvalho C, Rodriguez-Coleman H, Slakter J, et al Retinal angiomatous proliferation in age–related macular degeneration. Retina. 2012;32:416–34. 11642370 10.1097/00006982-200110000-00003

[3] Miere A, Querques G, Semoun O, Amoroso F, Zambrowski O, Chapron T, et al Optical coherence tomography angiography changes in early type 3 neovascularization after anti-vascular endothelial growth factor treatment. Retina. 2017;37(10):1873–9. 10.1097/IAE.0000000000001447 28079756

[4] Tan A, Dansingani KK, Yannuzzi LA, Sarraf D, Freund KB. Type 3 neovascularization imaged with cross-sectional and en face optical coherence tomography angiography. Retina. 2017;37(2):234–46. 10.1097/IAE.0000000000001343 27749497

[5] Shimada H, Kawamura A, Mori R, Yuzawa M. Clinicopathological findings of retinal angiomatous proliferation. Graefes Arch Clin Exp Ophthalmol. 2007;245(2):295–300. 10.1007/s00417-006-0367-6 16738855

[6] Bhavsar KV, Jia Y, Wang J, Patel RC, Lauer AK, Huang D, et al Projection-resolved optical coherence tomography angiography exhibiting early flow prior to clinically observed retinal angiomatous proliferation. Am J Ophthalmol. 2017;8:53–7. 10.1016/j.ajoc.2017.10.001 PMC5731673 29260118

[7] Sacconi R, Sarraf D, Garrity S, Freund KB, Yannuzzi LA, Gal-Or O, et al Nascent Type 3 Neovascularization in Age-Related Macular Degeneration. Ophthalmol Retina. 2018. 10.1016/j.oret.2018.04.016 31047548

[8] Gao SS, Jia Y, Zhang M, Su JP, Liu G, Hwang TS, et al Optical coherence tomography angiography. Invest Ophthalmol Vis Sci. 2016;57(9):OCT27–OCT36. 10.1167/iovs.15-19043 27409483 PMC4968919

[9] Louzada RN, Talisa E, Adhi M, Novais EA, Durbin MK, Cole E, et al Optical coherence tomography angiography artifacts in retinal pigment epithelial detachment. Can J Ophthalmol. 2017;52(4):419–24. 10.1016/j.jcjo.2016.12.012 28774527

[10] Zhang M, Hwang TS, Campbell JP, Bailey ST, Wilson DJ, Huang D, et al Projection-resolved optical coherence tomographic angiography. Biomed Opt Express. 2016;7(3):816–28. 10.1364/BOE.7.000816 27231591 PMC4866458

[11] Hwang TS, Zhang M, Bhavsar K, Zhang X, Campbell JP, Lin P, et al Visualization of 3 distinct retinal plexuses by projection-resolved optical coherence tomography angiography in diabetic retinopathy. JAMA Ophthalmol. 2016;134(12):1411–9. 10.1001/jamaophthalmol.2016.4272 27812696 PMC5805384

[12] Chu S, Nesper PL, Soetikno BT, Bakri SJ, Fawzi AA. Projection-Resolved OCT Angiography of Microvascular Changes in Paracentral Acute Middle Maculopathy and Acute Macular Neuroretinopathy. Invest Ophthalmol Vis Sci. 2018;59(7):2913–22. 10.1167/iovs.18-24112 30025133 PMC5989859

[13] Nesper PL, Soetikno BT, Treister AD, Fawzi AA. Volume-Rendered Projection-Resolved OCT Angiography: 3D Lesion Complexity Is Associated With Therapy Response in Wet Age-Related Macular Degeneration. Invest Ophthalmol Vis Sci. 2018;59(5):1944–52. 10.1167/iovs.17-23361 29677356 PMC5894925

[14] Bolz M, Schmidt-Erfurth U, Deak G, Mylonas G, Kriechbaum K, Scholda C. Optical coherence tomographic hyperreflective foci: a morphologic sign of lipid extravasation in diabetic macular edema. Ophthalmology. 2009;116(5):914–20. 10.1016/j.ophtha.2008.12.039 19410950

[15] Uji A, Murakami T, Nishijima K, Akagi T, Horii T, Arakawa N, et al Association between hyperreflective foci in the outer retina, status of photoreceptor layer, and visual acuity in diabetic macular edema. Am J Ophthalmol. 2012;153(4):710–7. e1. 10.1016/j.ajo.2011.08.041 22137207

[16] Ogino K, Murakami T, Tsujikawa A, Miyamoto K, Sakamoto A, Ota M, et al Characteristics of optical coherence tomographic hyperreflective foci in retinal vein occlusion. Retina. 2012;32(1):77–85. 10.1097/IAE.0b013e318217ffc7 21866075

[17] Chen KC, Jung JJ, Curcio CA, Balaratnasingam C, Gallego-Pinazo R, Dolz-Marco R, et al Intraretinal hyperreflective foci in acquired vitelliform lesions of the macula: clinical and histologic study. Am J Ophthalmol. 2016;164:89–98. 10.1016/j.ajo.2016.02.002 26868959

[18] Christenbury JG, Folgar FA, O'Connell RV, Chiu SJ, Farsiu S, Toth CA. Progression of intermediate age-related macular degeneration with proliferation and inner retinal migration of hyperreflective foci. Ophthalmology. 2013;120(5):1038–45. 10.1016/j.ophtha.2012.10.018 23352193 PMC3640702

[19] Ho J, Witkin AJ, Liu J, Chen Y, Fujimoto JG, Schuman JS, et al Documentation of intraretinal retinal pigment epithelium migration via high-speed ultrahigh-resolution optical coherence tomography. Ophthalmology. 2011;118(4):687–93. 10.1016/j.ophtha.2010.08.010 21093923 PMC3070873

[20] Miura M, Makita S, Sugiyama S, Hong Y-J, Yasuno Y, Elsner AE, et al Evaluation of intraretinal migration of retinal pigment epithelial cells in age-related macular degeneration using polarimetric imaging. Sci Rep. 2017;7(1):3150 10.1038/s41598-017-03529-8 28600515 PMC5466639

[21] Hemeida TS, Keane PA, Dustin L, Sadda SR, Fawzi AA. Long-term visual and anatomical outcomes following anti-VEGF monotherapy for retinal angiomatous proliferation. Br J Ophthalmol. 2010;94(6):701–5. 10.1136/bjo.2009.167627 19854733 PMC2878743

[22] Jia Y, Tan O, Tokayer J, Potsaid B, Wang Y, Liu JJ, et al Split-spectrum amplitude-decorrelation angiography with optical coherence tomography. Opt Express. 2012;20(4):4710–25. 10.1364/OE.20.004710 22418228 PMC3381646

[23] Campbell J, Zhang M, Hwang T, Bailey S, Wilson D, Jia Y, et al Detailed vascular anatomy of the human retina by projection-resolved optical coherence tomography angiography. Sci Rep. 2017;7:42201 10.1038/srep42201 28186181 PMC5301488

[24] Garrity ST, Iafe NA, Phasukkijwatana N, Chen X, Sarraf D. Quantitative analysis of three distinct retinal capillary plexuses in healthy eyes using optical coherence tomography angiography. Invest Ophthalmol Vis Sci. 2017;58(12):5548–55. 10.1167/iovs.17-22036 29075766

[25] Iafe NA, Phasukkijwatana N, Chen X, Sarraf D. Retinal capillary density and foveal avascular zone area are age-dependent: quantitative analysis using optical coherence tomography angiography. Invest Ophthalmol Vis Sci. 2016;57(13):5780–7. 10.1167/iovs.16-20045 27792812

[26] Querques G, Souied EH, Freund KB. How has high-resolution multimodal imaging refined our understanding of the vasogenic process in type 3 neovascularization? Retina. 2015. 10.1097/IAE.0000000000000487 25621948

[27] Su D, Lin S, Phasukkijwatana N, Chen X, Tan A, Freund KB, et al An updated staging system of type 3 neovascularization using spectral domain optical coherence tomography. Retina. 2016;36:S40–S9. 10.1097/IAE.0000000000001268 28005662

[28] Wang J, Zhang M, Hwang TS, Bailey ST, Huang D, Wilson DJ, et al Reflectance-based projection-resolved optical coherence tomography angiography. Biomed Opt Express. 2017;8(3):1536–48. 10.1364/BOE.8.001536 28663848 PMC5480563

[29] Nesper PL, Lutty GA, Fawzi AA. Residual choroidal vessels in atrophy can masquerade as choroidal neovascularization on optical coherence tomography angiography: introducing a clinical and software approach. Retina. 2018;38(7):1289–300. 10.1097/IAE.0000000000001863 29059100 PMC5910298

